# Supplementary material for: Study of Structural and Optoelectronic Properties of Thin Films Made of a Few Layered WS2 Flakes
Source: Materials (Basel). 2020 Nov 24;13(23):5315. doi: 10.3390/ma13235315 (PMC7727660; doi:10.3390/ma13235315)
Supplement: Supplementary file 1 [file materials-13-05315-s001.pdf]

materials

MDPI

Supplementary Materials

Study of Structural and Optoelectronic Properties of Thin Films Made of a Few Layered WS_2 Flakes

Anna Łapińska [1,*], Michał Kuźniewicz [1], Arkadiusz P. Gertych [1], Karolina Czerniak-Łosiewicz [1], Klaudia Żerańska-Chudek [1], Anna Wróblewska [1], Michał Świniarski [1], Anna Dużyńska [1], Jarosław Judek [1], Mariusz Zdrojek [1]

[1] Faculty of Physics, Warsaw University of Technology, Koszykowa 75, 00-662 Warsaw, Poland; michal.kuzniewicz.stud@pw.edu.pl (M.K.); Arkadiusz.Gertych@pw.edu.pl (A.P.G.); karolina.czerniak@pw.edu.pl (K.C.-L.); Klaudia.Zeranska@pw.edu.pl (K.Z.-C.); anna.wroblewska@pw.edu.pl (A.W.); Michal.Swiniarski@pw.edu.pl (M.S.); anna.duzynska@pw.edu.pl (A.D.); jaroslaw.judek@pw.edu.pl (J.J.); mariusz.zdrojek@pw.edu.pl (M.Z.)

* Correspondence: anna.lapinska@pw.edu.pl

Received: 21 October 2020; Accepted: 19 November 2020; Published: date

1. Atomic Force Microscopy (AFM) and Scanning Electron Microscopy (SEM) Measurements Details.

Atomic Force Microscopy (AFM, Brucker Icon, Billerica, MA, USA) measurements were performed using tapping mode and OTESPA-R3 tips. The reported thickness profiles (as well as flakes and film) were extracted measuring the difference between substrate and edges of flake/film height as it is shown in Figures S2 and S3. The thickness of the flakes was calculated measuring direct height differences between substrate and the flake. Considering the film thickness, the average value of the measured film height (due to its roughness) and the substrate was used.

The RMS value of the film (Figure S1b) was calculated using the built-in Gwyddion software option (Gwyddion – Free SPM (AFM, SNOM/NSOM, STM, MFM) data analysis software, version 2.56, Department of Nanometrology, Czech Metrology Institute, Jihlava, Czech Republic). The RMS (σ) value was then extracted based on the following equations:

$$\sigma = \mu_2^{1/2} \quad (1)$$

$$\mu_i = \frac{1}{N}\sum_{n=1}^{N}(z_n - \bar{z})^i \quad (2)$$

$$\gamma_1 = \frac{\mu_3}{\mu_2^{3/2}} \quad (3)$$

where: γ_1–skewness, γ_2–kurtosis

The electron microscopy (SEM, eLine Plus, GmbH, Dortmund, Germany) measurements were carried out using InLens detector. The electron accelerating voltage (EHT) was set at 10 kV, the work distance (WD) was 11.2 mm, giving the ×30,000 magnitude. No sputtering above the sample was used.

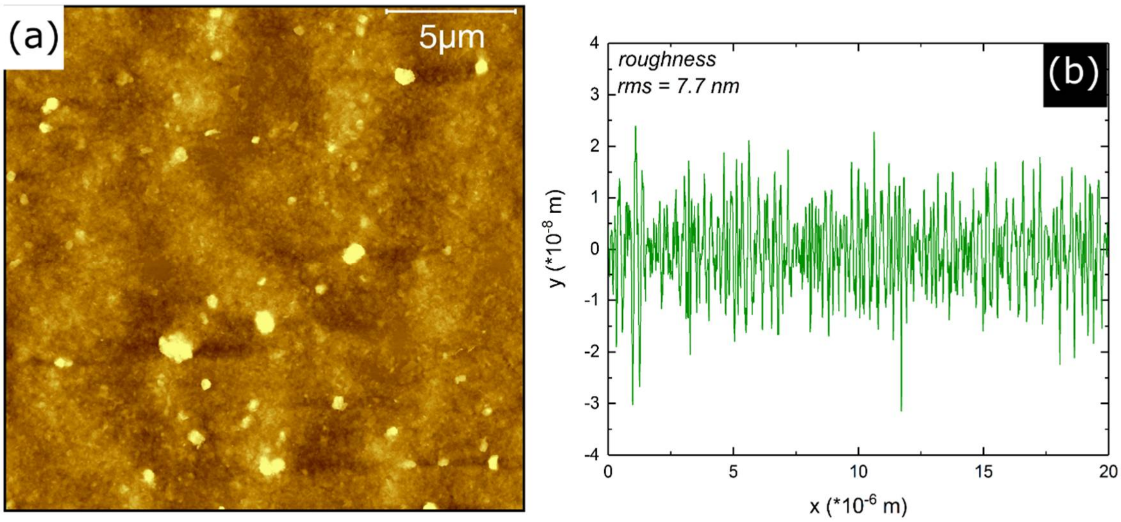

Figure S1. (**a**) AFM scan of WS_2 thin film, (**b**) roughness graph with rms parameter calculated.

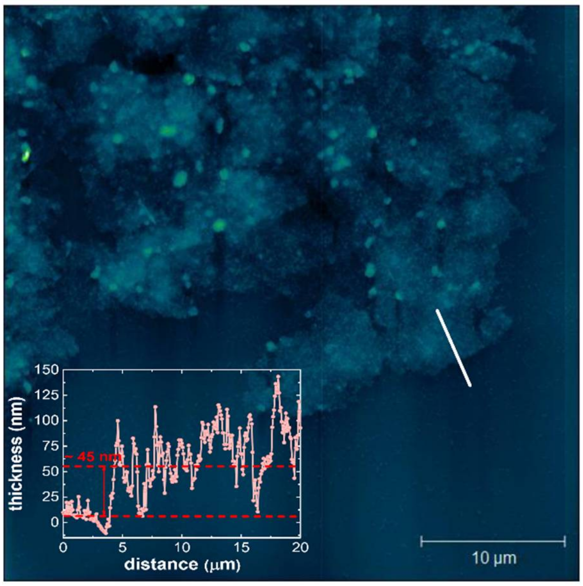

Figure S2. AFM scan with thickness profile'inset. White line indicates the measurement area, which then the profile had been extracted. Due to high roughness of the film, the thickness profile had been determined using average value.

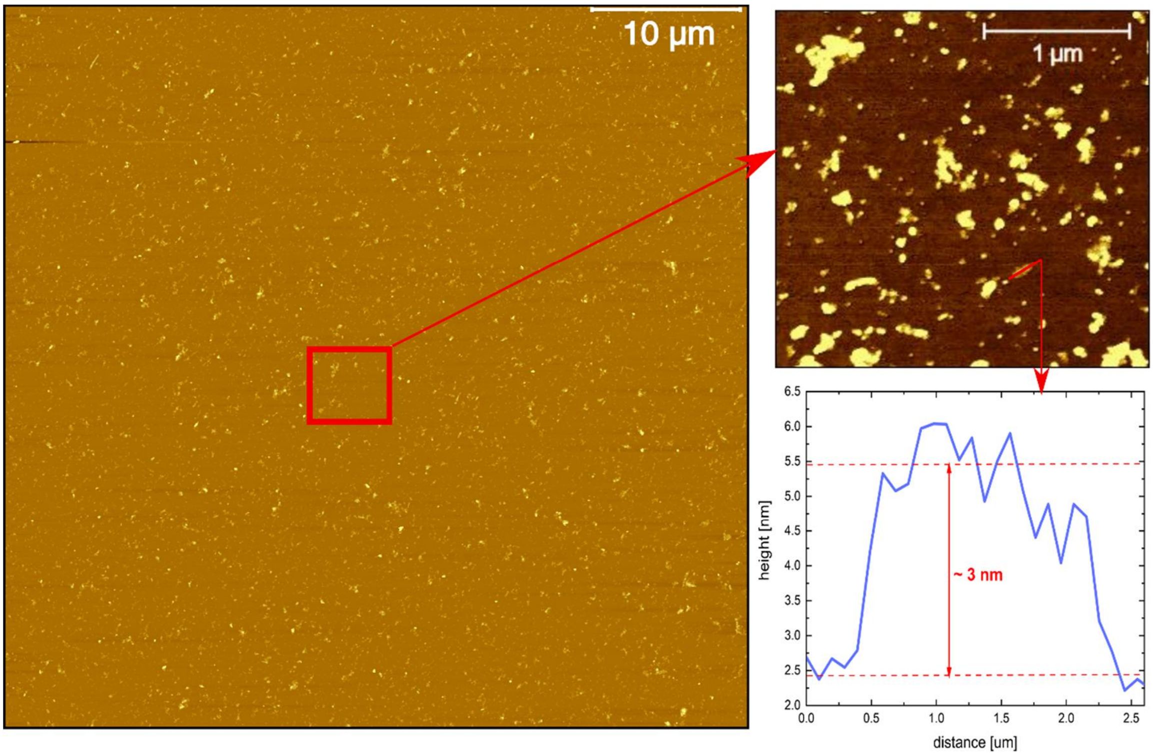

Figure S3. AFM high-resolution scan (50 × 50 µm, 5008 lines) of individual flakes from few-layer WS_2 suspension that is used for film production. Flake thickness distribution is derived from measurement of single flake measurement from that scan by divided it into smaller scans as it is shown in the upper, right corner (the size of this scan is 2.5 × 2.5 µm). The red line in this scan corresponds to the bottom thickness profile of marked, individual flake.

2. Other 2D Exfoliated Materials.

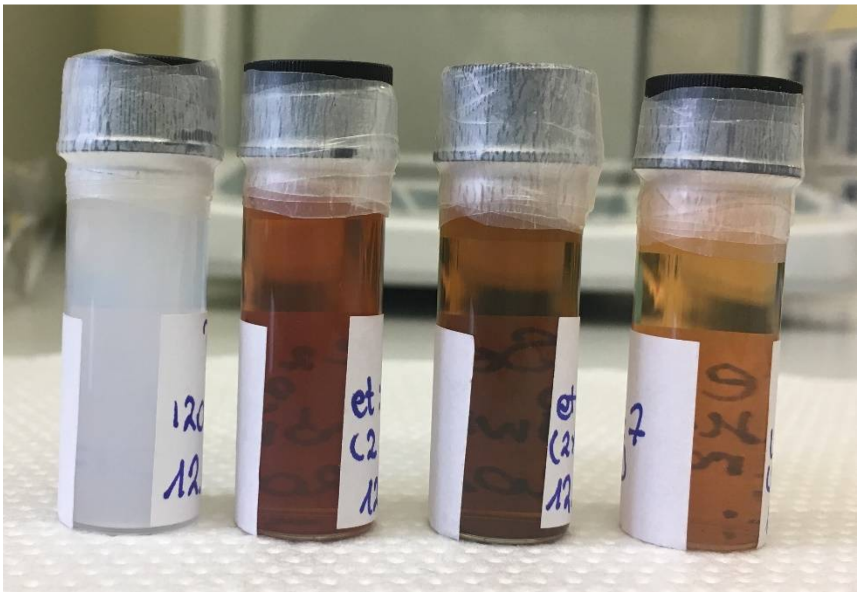

Figure S4. Suspension made via "green" liquid exfoliation (DI water/IPA) of (from left) hBN, $SnSe_2$, GeSe, GeS.

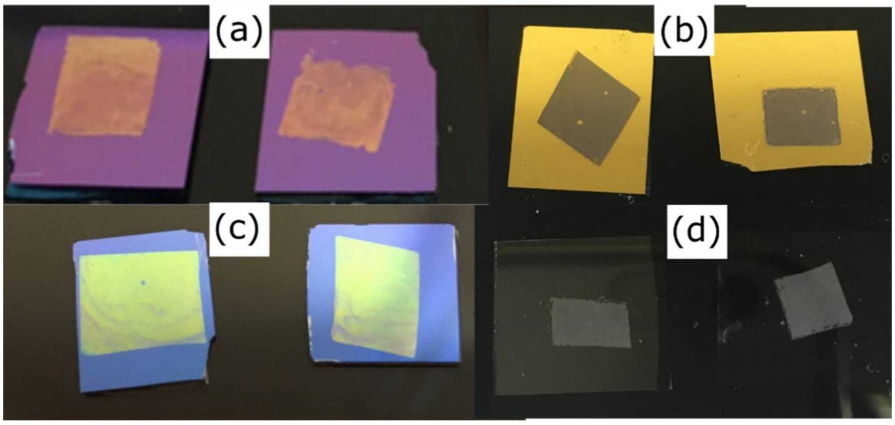

Figure S5. Thin film made via vacuum filtration transferred on different substrates: (**a**) $SnSe_2$ on SiO_2/Si substrate, (**b**) MoS_2 on gold substrate, (**c**) GeSe on SiO_2/Si substrate,(**d**) MoS_2 on glass substrate.

Publisher's Note: MDPI stays neutral with regard to jurisdictional claims in published maps and institutional affiliations.

© 2020 by the authors. Licensee MDPI, Basel, Switzerland. This article is an open access article distributed under the terms and conditions of the Creative Commons Attribution (CC BY) license (http://creativecommons.org/licenses/by/4.0/).
